# Supplementary material for: Probiotic Enrichment and Reduction of Aflatoxins in a Traditional African Maize-Based Fermented Food
Source: Nutrients. 2019 Jan 25;11(2):265. doi: 10.3390/nu11020265 (PMC6412935; doi:10.3390/nu11020265)
Supplement: Supplementary file 1 [file nutrients-11-00265-s001.pdf]

Supplementary Materials

1. fit

Variables

Ymin	Ymax	LogEC50-	LogEC50-2	nH1	nH2	Frac	RSS	sumX^2
6267.199287	66988.7107	-0.149639	0.4977961	-26.01181739	-11.59861972	0.986802	13.43	1835211.15

Equation 1:

$$y = y_{min} + (y_{max} - y_{min})\left(\frac{(Frac)}{1+10^{((LogEC50_1 - x)\,nH_1)}} + \frac{(1-Frac)}{1+10^{((LogEC50_2 - x)\,nH_2)}}\right)$$

L. rhamnosus yoba 2012 (OD)	Residual aflatoxin (arbitruary units)	Fit	X^2	Residuals
1.037	6282.167	6268.737204	180.3594079	-13.43
0.55	6556.75	6550.167806	43.32528344	-6.5822
0.289	7115	7003.933866	12335.68618	-111.07
0.156	7251	7074.799473	31046.62567	-176.2
0.08	6767	7214.509917	200265.1257	447.51
0.045	7580.5	7441.163661	19414.6155	-139.34
0.013	7365	7924.730594	313298.3381	559.73
0.005	7787	8120.203389	111024.4982	333.2
0.002	8053.333	8204.229903	22769.87548	150.9
0	8670	8263.851748	164956.4023	-406.15
0.11	7338.222	7129.603583	43521.64386	-208.62
0.057	7404.5	7339.999213	4160.351549	-64.501
0.033	7994.5	7578.535943	173026.0968	-415.96
0.016	7585.111	7861.000399	76114.96075	275.89
0.007	7676	8067.592746	153344.8789	391.59
0	8411	8263.851748	21652.60797	-147.15
1.013	6277	6269.22958	60.37941936	-7.7704
0.776	6281.3	6297.789258	271.8956178	16.489
0.72	6315	6323.791933	77.29808663	8.7919
0.521	6611.3	6614.30695	9.041750704	3.007
0.391	6886	6888.606619	6.794463393	2.6066
0.266	6960	7018.80297	3457.789248	58.803
0.2	6927	7050.77091	15319.23828	123.77
0.192	6921.5	7054.442103	17673.60279	132.94
0.1	6943.5	7151.271091	43168.8262	207.77
0.091	7115	7175.878209	3706.156272	60.878
0.076	7482	7231.428158	62786.24815	-250.57
0.033	7994.5	7578.535943	173026.0968	-415.96
0	8674.33	8263.851748	168492.3951	-410.48

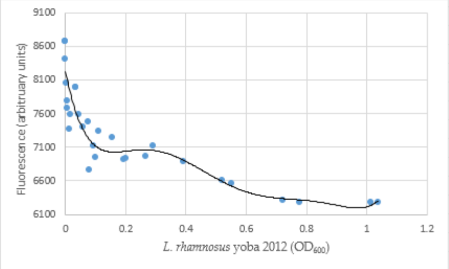

***L. rhamnosus* yoba 2012 – aflatoxin B1 binding curve**

2. residuals

OD	residuals
1.037	-12.876
0.55	-8.3178
0.289	-114.69
0.156	-187.82
0.08	451.8
0.045	-118.52
0.013	573.768
0.005	330.676
0.002	139.061
0	-425.35
0.11	-214.16
0.057	-49.309
0.033	-391.67
0.016	293.717
0.007	394.236
0	-166.35
1.013	-7.389
0.776	11.1091
0.72	0.79216
0.521	5.95324
0.391	12.1015
0.266	52.4212
0.2	112.151
0.192	121.053
0.1	204.89
0.091	60.9094
0.076	-244.55
0.033	-391.67
0	-429.68

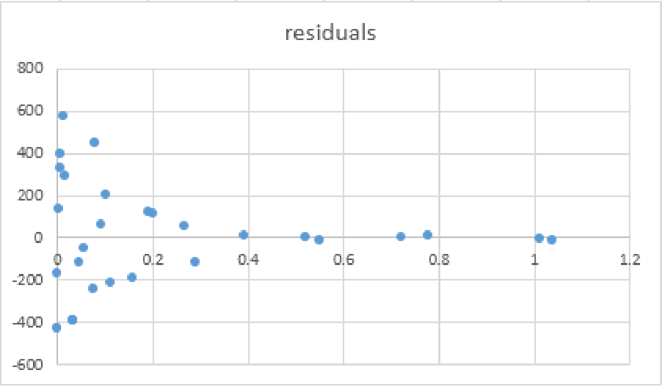

3. Calculated r2

L. rhamr	Residual aflatoxin (arbitruary	Fit	(Residual aflatoxin - Fit) squared	aflatoxin -mean of residual aflatoxin)
1.037	6282.167	6268.74	180.3594079	1002944.924
0.55	6556.75	6550.17	43.32528344	528366.716
0.289	7115	7003.93	12335.68618	28438.90298
0.156	7251	7074.8	31046.62567	1065.263804
0.08	6767	7214.51	200265.1257	266915.215
0.045	7580.5	7441.16	19414.6155	88126.82184
0.013	7365	7924.73	313298.3381	6619.713321
0.005	7787	8120.2	111024.4982	253372.9212
0.002	8053.333	8204.23	22769.87548	592429.8091
0	8670	8263.85	164956.4023	1921998.543
0.11	7338.222	7129.6	43521.64386	2979.371648
0.057	7404.5	7340	4160.351549	14607.53136
0.033	7994.5	7578.54	173026.0968	505324.2438
0.016	7585.111	7861	76114.96075	90885.74103
0.007	7676	8067.59	153344.8789	153947.6414
0	8411	8263.85	21652.60797	1270944.224
1.013	6277	6269.23	60.37941936	1013320.827
0.776	6281.3	6297.79	271.8956178	1004682.227
0.72	6315	6323.79	77.29808663	938260.3099
0.521	6611.3	6614.31	9.041750704	452038.8963
0.391	6886	6888.61	6.794463393	158116.2807
0.266	6960	7018.8	3457.789248	104741.8006
0.2	6927	7050.77	15319.23828	127190.9336
0.192	6921.5	7054.44	17673.60279	131144.2058
0.1	6943.5	7151.27	43168.8262	115694.1171
0.091	7115	7175.88	3706.156272	28438.90298
0.076	7482	7231.43	62786.24815	39347.33256
0.033	7994.5	7578.54	173026.0968	505324.2438
0	8674.33	8263.85	168492.3951	1934023.184
Mean	7283.638379	Total	1835211.154	13281290.84
	The percentage of total variation not		0.13818018	
		R2 =	0.86181982	

4. Total binding sites per bacteri

calculated from the minimum value for Y (Ymin) using the equation from the standard curve below.

Ymin	6267.2	
n (*L. rhamnosus* yoba 2012 corresponding to Ymin) (OD600)	1	8.0×10^{8} cfu (average of three independent plate counts)
Aflatoxin B_1 used in the experiment (µg ml⁻	1	

Data used for development of standard curve

flatoxin B_1 (µg ml⁻	Fluorescence (arbitrary uits) trail 1	trail 2	trail 3	Mean
10	11134	12495	12709	12112.7
5	9142	9325	9234	9233.67
1	6767	7281	7314	7120.67
0.5	6134	6533	6608	6425
0.01	6366	6316	6308	6330
0	5893	6029	6165	6029

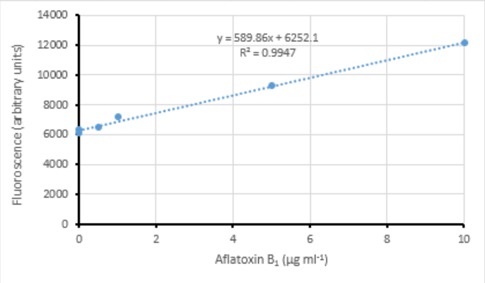

Standard curve of aflatoxin B1 (µg ml⁻¹)

From the standard curve, the concentration of free aflatoxin B_1 = (Ymin − 6252.1)/589.86

'ree aflatoxin B_1 (unbound	0.025598086
L. rhamnosus yoba 2012 corresponding to	800000000
bound aflatoxin (Bmax) (µ	0.974401914
Avogadros constant (mol⁻¹)	6.022E+23

The total number of aflatoxin binding sites per bacterium (M) =

M	2348642.463
